# Recent progress in modeling and treating diabetes using stem cell-derived islets

**DOI:** 10.1093/stcltm/szae059

**Published:** 2024-08-19

**Authors:** Marlie M Maestas, Maggie H Bui, Jeffrey R Millman

**Affiliations:** Roy and Diana Vagelos Division of Biology and Biomedical Sciences, Washington University School of Medicine, St. Louis, MO 63110, United States; Division of Endocrinology, Metabolism, and Lipid Research, Washington University School of Medicine, St. Louis, MO 63110, United States; Roy and Diana Vagelos Division of Biology and Biomedical Sciences, Washington University School of Medicine, St. Louis, MO 63110, United States; Roy and Diana Vagelos Division of Biology and Biomedical Sciences, Washington University School of Medicine, St. Louis, MO 63110, United States; Division of Endocrinology, Metabolism, and Lipid Research, Washington University School of Medicine, St. Louis, MO 63110, United States; Department of Biomedical Engineering, Washington University in St. Louis, St. Louis, MO 63110, United States

**Keywords:** differentiation, pancreatic differentiation, pluripotent stem cells, diabetes modeling, transplantation, diabetes, cellular therapy, immunosuppression

## Abstract

Stem cell-derived islets (SC-islets) offer the potential to be an unlimited source of cells for disease modeling and the treatment of diabetes. SC-islets can be genetically modified, treated with chemical compounds, or differentiated from patient derived stem cells to model diabetes. These models provide insights into disease pathogenesis and vulnerabilities that may be targeted to provide treatment. SC-islets themselves are also being investigated as a cell therapy for diabetes. However, the transplantation process is imperfect; side effects from immunosuppressant use have reduced SC-islet therapeutic potential. Alternative methods to this include encapsulation, use of immunomodulating molecules, and genetic modification of SC-islets. This review covers recent advances using SC-islets to understand different diabetes pathologies and as a cell therapy.

## Graphical Abstract

**Graphical Abstract:**
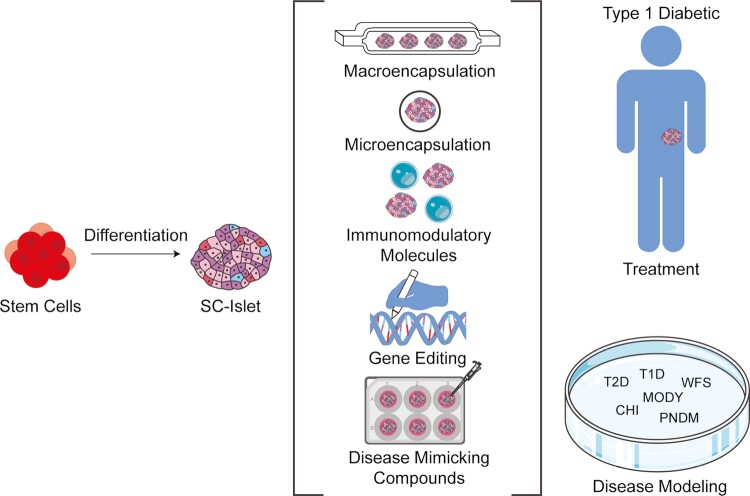

**Significance Statement**

Stem cell-derived islets (SC-islets) are a promising technology enabling cell replacement therapy and development of improved disease modeling of diabetes. This review highlights progress made in the field, including SC-islet differentiation, encapsulation, immunomodulatory molecules, gene editing, and disease-mimicking approaches. Continued development of SC-islets will lead to new treatments for many different types of diabetes.

## Introduction

The differentiation of stem cell-derived islets (SC-islets) can be used to model and treat diabetes. Diabetes affects approximately 537 million adults worldwide.^1^ This disease is due to loss or improper function of pancreatic β-cells, which are important to regulate blood glucose levels.^2,3^ The mechanism of β-cell death or dysfunction in different forms of diabetes is unknown. To better investigate diabetes pathology, SC-islets provide a platform for disease modeling and drug discovery. Avenues of study include using patient-derived SC-islets to understand the pathophysiology of monogenic forms of diabetes, genetically altering SC-islets to recapitulate patient mutations, or exposing SC-islets to compounds that induce a diabetic phenotype.

The current standard of care for diabetes requires a combination of rigorous monitoring of blood glucose levels, exogenous insulin injections, and lifestyle changes.^2^ However, this treatment does not mitigate micro- and macrovascular changes that lead to end organ damage and the development of secondary complications of diabetes such as diabetic retinopathy, neuropathy, nephropathy, and coronary artery disease, all of which decrease patient quality of life.^2-5^ As such, a therapy capable of mimicking islet function and replacing the lost or dysfunctional β-cells would be a breakthrough in diabetes treatment. Currently, SC-islets are being pioneered for this purpose as they can be produced at scale and have many of the defining characteristics found within primary human islets. While much work has been done to produce and facilitate SC-islet transplantation, challenges such as finding an alternative to immunosuppressant use are still being actively investigated.^6^ This review will summarize recent papers using SC-islets for disease modeling and for improving transplantation by creating less immunogenic SC-islets.

## Generating stem cell-derived islets

### Current differentiation methods

**Figure 1.**
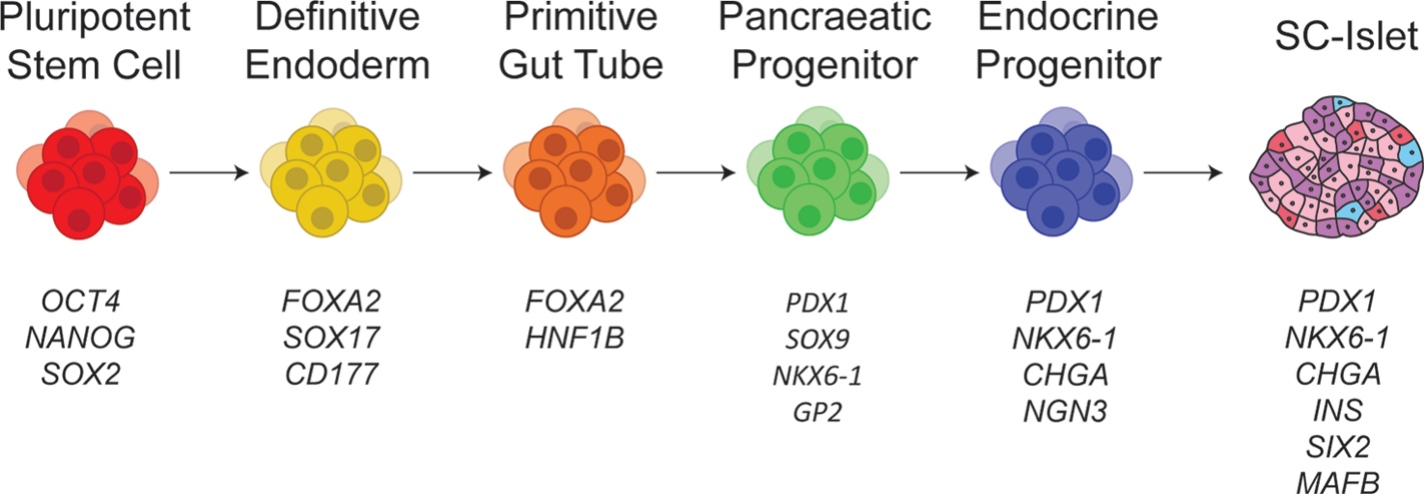
Representative diagram of the differentiation of SC-islets. While the differentiation protocols for SC-islets can differ in the small molecules or growth factors used, they all go through similar developmental stage progression.^10-19^ The nomenclature for each stage can vary between protocols. The first stage is the definitive endoderm, characterized by the induction of FOXA2, SOX17, and CD177. FOXA2 and HNF1B are markers of the next stage, the primitive gut tube. Pancreatic progenitor cells express PDX1, SOX9, NKX6-1, and GP2. In addition to PDX1 and NKX6-1, endocrine progenitor cells express CHGA and NGN3. Common markers for SC-islets are INS, SIX2, GCG, SST, and NKX6-1.

The foundation of modern SC-islet protocols stems from understanding the endogenous embryonic development of pancreatic islets. In humans, the definitive endoderm (DE) is the first stage of pancreatic islet development.^7^ This can be replicated by inducing TGFβ^8^ and Wnt signaling^9^ in stem cells, creating FOXA2 and SOX17 positive cells (Figure 1). Next is the formation of the primitive gut tube by activation of RTK signaling.^10^ The cells are then further differentiated into the posterior foregut and then pancreatic progenitor cells using various combinations of RTK activation, retinoic acid, hedgehog signaling inhibition,^10^ bone morphogenetic protein (BMP) signaling activation,^11,12^ and PKC signaling activation.^13,14^ The pancreatic progenitor cells express NKX6-1^12^ (Figure 1). The addition of thyroid hormones and retinoic acid, and inhibition of hedgehog, TGFβ, and Notch^13^ or BMP^14^ signaling create pancreatic endocrine cells. The first stem cell-derived endocrine precursor cells contained insulin but had low secretion in response to glucose,^10^ prompting many groups to pursue functional improvement of these cells. Interestingly, Kroon et al.^11^ transplanted SC-derived pancreatic progenitor cells into diabetic mouse models and found that after transplantation, the cells secreted insulin and significantly lowered blood glucose, illustrating the functional maturation of the SC-derived pancreatic progenitor cells in vivo. The field took another step forward when the production of similarly functional cells was achieved solely in vitro.^13-15^ The modulation of TGFβ signaling in later stages further improved the maturation of SC-endocrine cells leading to a dynamic response to glucose.^16^ Additional studies have targeted the cytoskeleton,^17^ used aggregation methods,^16,18^ or circadian entrainment^19^ to improve SC-islets maturation in vitro.

### Single-cell profiling

While SC-islets are similar to primary islets, they neither fully recapitulate primary islet function^20^ nor express all the same markers.^21^ This led to the investigation of islet composition using single-cell sequencing. The pancreatic islet of Langerhans is a heterogeneous tissue composed of multiple cell types, including β-, α-, δ-, ε-cells, and pancreatic polypeptide cells.^22-24^ Transcriptional profiling of cell types has indicated that *MAFA* and *SIX3*, maturation markers expressed in primary human islets, are not expressed in SC β-cells.^21,25^ After transplantation into a diabetic mouse model, NOD.Cg-*Prkdc*^*scid*^*Il2*^*rgtm*1*Wjl*^/SzJ (NSG), single-cell sequencing revealed the expression of *MAFA*, *UCN3*, and *G6PC2*^26^ increased with time,^27^ although the mechanism for this in vivo maturation remains unknown. Single-cell sequencing data also identified a surface marker CD49a, which could be used to sort for SC β-cells.^25^

SC-islet differentiations produce stem cell-derived enterochromaffin-like cells (SC-EC cells),^25^ regardless of the differentiation protocol.^21^ These off-target SC-EC cells are an enteroendocrine cell type found within the intestine^28^ and are not found in primary human islets. *TPH1*, *DDC*, and *LMX1A* are key markers used to identify SC-EC cells.^25^ The gene expression profiles of SC β-cells and SC-EC cells led to the hypothesis that these cell types arise from a common progenitor but acquire different expression patterns at the very end of the differentiation. More recently, the addition of single nuclei assay for transposase-accessible chromatin sequencing (snATAC-seq) has provided evidence indicating that SC β-cells and SC-EC cells are more related to each other than previously thought.^29,30^ In addition, snATAC-seq has allowed for the identification of candidate genes to improve the differentiation of SC-islets.^29^

## Disease modeling

**Figure 2.**
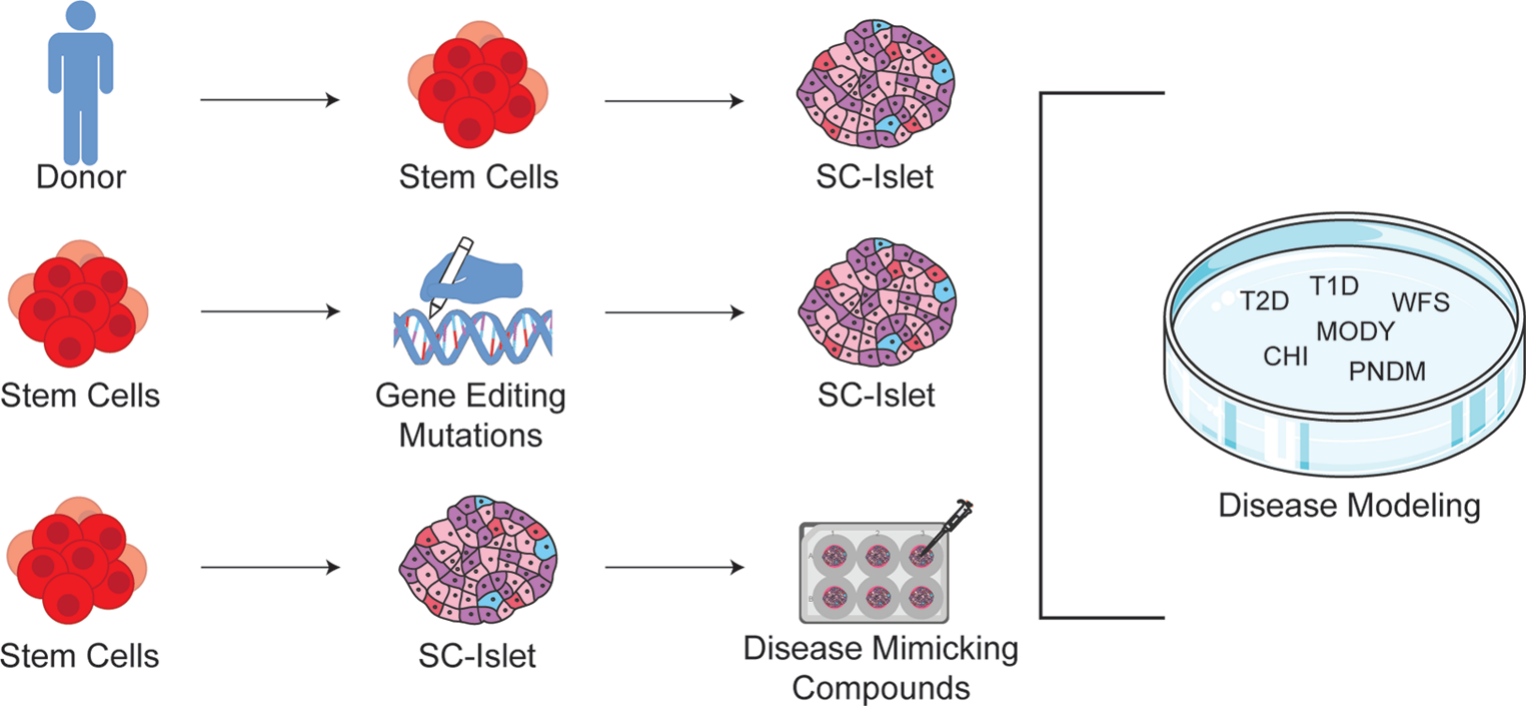
SC-islets as a disease model for diabetes. Here, 3 methods are described using SC-islets to model different forms of diabetes such as Maturity-onset diabetes of the young (MODY), Wolfram Syndrome (WFS), permanent neonatal diabetes (PNDM), congenital hyperinsulinemia (CHI), type 1 diabetes (T1D), or type 2 diabetes (T2D). The first method is through the induction of pluripotent stem cells from patients with these forms of diabetes. These patient-derived stem cells are then differentiated into SC-islets. The second method is by introducing diabetes-associated mutations into stem cells. Researchers then differentiate the stem cells into stem cell-derived endocrine cells and compare the mutation with wild-type/isogenic control. The third way is to differentiate SC-islets using nondiabetic stem cells and culture them with compounds that mimic the diabetic environment.

Mouse models are commonly used to model diabetes. However, mouse islets have distinct differences compared to human islets.^31,32^ Primary human islets have a lower proportion of β- to α-cells than mouse islets.^33^ Spatially, mouse islets have a core composed of β-cells, while human islets have β-cells distributed throughout the islet,^33^ which affects function.^34^ Isolating human islets from deceased donors provides another cell source to study diabetes; however, these cells cannot be cultured in vitro for an extended period, a limited supply of donors are available, and there is donor-to-donor variability. SC-islets have provided a valuable resource by being readily available and plentiful, and they can be cultured for extended periods. Here, recent literature is highlighted that use genetic and chemical methods to model diabetes using SC-islets (Figure 2). This review mainly focuses on recent publications to provide a contemporary perspective on disease modeling and to build upon previously published reviews.^35-39^

### Patient-derived or genetically edited SC-islets

Various studies use human induced pluripotent stem cells (iPSCs) derived from patients with diabetes or CRISPR/Cas9 to introduce a mutation into human pluripotent stem cells (hPSC). iPSC- or hPSC-derived SC-islets are used to investigate many diabetes-associated diseases such as Maturity-onset diabetes of the young (MODY), Wolfram Syndrome (WFS), permanent neonatal diabetes (PNDM), congenital hyperinsulinemia (CHI), type 1 diabetes (T1D), and type 2 diabetes (T2D; Figure 2).

Induced pluripotent stem cells have been derived from a variety of MODY patients.^40-42^*HNF1A* mutations, which cause MODY-3, a monogenic form of diabetes, have been studied using SC-islets.^43-46^ Recent studies used iPSCs from HNF1A-MODY patients or CRISPR/Cas9 to target *HNF1A* and differentiate the stem cells into SC-derived endocrine cells. These *HNF1A* mutations caused the differentiation to favor an α-cell signature, reduced insulin secretion,^43,44^ decreased calcium levels, changed morphology of insulin granules,^43^ and reduced metabolic function^44^ in SC-derived endocrine cells. In opposition, a different mutation in *HNF1A* induced insulin hypersecretion and increased calcium signaling.^45^ A common truncated mutation in *HNF1A* induced disruptions to the differentiation by interacting with *HNF1B*, leading to reduced *PDX1* and *NKX6.1* expression in pancreatic progenitors.^46^ These studies have added to the existing literature on pathogenesis of MODY-3 and emphasized the importance of studying different mutations within the *HNF1A* gene.

WFS is caused by mutations in the *WFS1* gene, which can lead to the development of diabetes mellitus and optic nerve atrophy.^47^ iPSCs from patients with WFS are used to study the diabetic aspect of this syndrome.^48^ A study investigated a mutation in the *WFS1* gene with high prevalence in the Ashkenazi Jewish population. iPSCs were produced from 2 WFS patients with different variants, and differentiated into SC-islets.^49^ The patient-derived SC-islets had different effects on cell type proportions, although both variants reduced insulin secretion in response to glucose. The depleted insulin secretion was improved with the administration of Tauroursodeozycholic acid (TUDCA) and 4-phenyl butyric acid (4-PBA), molecular chaperones that improve protein folding. This data was used as the basis for an ongoing clinical trial with AMX0035, which reported improvements in the glycemic control and vision of WFS patients.^50^ Another study used CRISPR/Cas9 to knockout (KO) *WFS1* in hPSCs. The authors found that *WFS1* KO SC-islets had downregulated translational signaling, which can be indicative of integrated stress response (ISR) activation.^51^ Use of the ISR inhibitor, ISRIB, decreased stress granules and apoptosis in *WFS1* KO SC-islets compared to control cells. This previous study provides evidence that targeting ISR could treat WFS-associated diabetes. Another approach used CRISPR/Cas9 to correct *WFS1* variants in patient-derived iPSCs.^52^ The SC-islets derived from the corrected iPSCs had restored insulin secretion. Transplantation of the corrected SC-islets cured diabetic mice, indicating the promise of SC-islet autologous cell replacement therapy for WFS patients.

PNDM is found in babies under 6 months of age who present with hyperglycemic events.^53^ Mutations in *INS*, which codes for insulin, are present in patients with PNDM. Heterozygous *INS* mutations cause dedifferentiation of patient-derived SC-islets by upregulating ALDH1A3+/NKX6.1+ cell populations after transplantation into a mouse model.^54^ The heterozygous mutations^54^ and a proinsulin cysteine mutation^55^ in *INS* induced endoplasmic reticulum (ER) stress in SC-islets. An intronic *INS* mutation created ectopic splicing sites, leading to a splice isoform in the patient-derived SC-islets, and reduced C-peptide secretion.^56^ If an *INS* mutation is corrected, this can restore insulin secretion in vitro.^57^

T1D is an autoimmune disease in which T cells attack the pancreatic β-cells.^4,5^ A variety of differentiation protocols derived SC-islets from patient-derived iPSCs.^58-60^ These cells functioned similar to nondiabetic SC-islets. *TYK2*, a risk variant for T1D, has been knocked out in iPSCs using CRISPR/Cas9,^61^ leading endocrine progenitor cells to have increased KRAS and reduced *NEUROG3*, *NKX6.1*, and *NKX2-2* expression. Interestingly, when SC-islets were treated with TNFα, the wild-type SC-islets had high levels of MHC Class I expression whereas the *TYK2* KO SC-islets had almost no expression. This provides a possible target to reduce the autoimmune attack on the pancreatic β-cells during T1D.

T2D is mainly characterized by impaired insulin sensitivity, leading to β-cell dysfunction.^3^ In Southwestern Native Americans, *KCNQ1*, located in an imprinted gene region, is associated with T2D.^62^ The authors targeted SNPs at the *KCNQ1* locus in hiPSCs derived from a Native American patient with T2D, resulting in a functional hemizygous deletion in *KCNQ1*. In endocrine progenitor cells, this deletion caused increased expression of *CDKN1C*, known to reduce β-cell mass, and decreased expression of *H19*, known to increase β-cell mass. Another T2D study used SC-islets to investigate *PAX4*^63^; variants in *PAX4* have been associated with T2D in East Asian populations. Knockout of *PAX4* in nondiabetic iPSCs did not inhibit the differentiation of these cells into SC-endocrine cells; however, there was increased expression of α-cell markers and decreased β-cell identity markers in SC-islets. The authors also used nondiabetic donor-derived iPSCs containing specific *PAX4* variants to differentiate SC-islets. These variants increased polyhormonal cells, lowered insulin content, and altered glycolysis. When they corrected the variant in iPSCs using CRISPR/Cas9, the phenotype was rescued. Parallel genomic platforms have also been developed to better interrogate T2D genetics with islet phenotype.^64^

There are multiple studies investigating CHI using stem cells.^65^ CHI can be caused by dysregulation of the K_ATP_-channel in β-cells. A recent publication used patient-derived iPSCs from a CHI patient with a mutation in the gene *ABCC8*, which encodes for the SUR1 protein,^66^ part of the K_ATP_-channel. This mutation increased the cell population, proliferation, and insulin secretion of SC-β cells. SUR1-mutated SC-islets induced hypoglycemia in transplanted mice. The in vivo and in vitro data from SC-islets recapitulate the phenotype seen in CHI patients with SUR1 mutations, leading to a promising disease model to further investigate this disease mechanism.

### Compounds to model diabetes in SC-islets

SC-islets can be treated with a variety of exogenous agents to recapitulate environmental elements of diabetes pathogenesis. Many studies have found increases in ER stress during diabetic conditions.^67,68^ Thapsigargin is a known chemical that induces ER stress by inhibiting the SERCA2b pump, causing changes in calcium levels.^69^ A study used thapsigargin, cytokine mix (IL1β, TNFα, and IFNy), or high glucose on SC-islets to study diabetes-associated stress.^70^ The authors compared the response of primary human islets and SC-islets to the previous conditions and found both exhibited increased genes associated with stress and immune interactions. Both primary and SC-islets also had decreased glucose-stimulated insulin secretion (GSIS) after treatement with the compounds. The authors then knocked down commonly upregulated genes under stress, *B2M*, *CDKN1*, *NLRC5*, and *XBP1* and found these genetic knock downs to reduce apoptosis in stressed SC-islets. Another study found that ER stress, in the form of thapsigargin, was essential to induce T-cell activation when co-culturing nondiabetic (ND) or T1D-derived SC-islets with autologous peripheral blood mononuclear cells.^71^ The authors also found the T-cell activation to be specific to co-cultures with stressed SC-β cells and not stressed SC-α cells, even though ER stress response was similar in both cell types. ER stress compounds have also been used to study the effects of drugs on pancreatic islets.^72^ The authors treated SC-islets with thapsigargin, or thapsigargin and imeglimin, an antidiabetic drug. Administration of thapsigargin alone increased apoptotic cells, while the addition of imeglimin reduced apoptosis. This study highlights the utility of SC-islets as a model system to study antidiabetic agents.

During the early stages of diabetes, immune cells infiltrate the pancreas and produce proinflammatory cytokines, such as IL-1, IFNα/γ, and TNFα. These cytokines can harm the pancreatic islet and induce cytocidal effects on isolated human islets.^73^ To determine if these cytokines had similar effects in SC-islets, a study compared SC-islets and primary human islets treated with cytokines.^74^ They found that IFNγ and IL1β increased apoptosis, induced inflammation- and ER stress-related gene expression, and decreased β-cell identity gene expression in both SC-islets and primary human islets. This data indicates that cytokine treatment induces a similar response in SC-islets and primary human islets.^74,75^ To understand inflammatory response during differentiation, inflammatory cytokines (IL1β, TNFα, and IFNy) were added to DE, multipotent pancreatic progenitor, and SC-β cells.^75^ They found increases in apoptosis in DE and SC-β cells, with a higher level of apoptosis in SC-β cells. There was no difference in nitric oxide (NO) levels in the supernatants of cytokine-treated SC-islets, which led to the hypothesis that the activation of apoptosis used a NO-independent pathway, such as JNK or p38 signaling, which were both upregulated under cytokine exposure. Another group knocked down *PTPN2,* a T1D susceptibility gene, in SC-islets, and found increased cytotoxicity when treated with IFNα or TNFα.^76^

Hyperglycemia and dysfunction of glucose uptake in the liver are features of T2D. To model this in vitro, a microfluidic system was used to co-culture hPSC-derived liver and islet^77^ cells. Treatment with high glucose for 5 days reduced oxygen consumption rate (OCR) and glucose transporter expression, reproducing features of T2D. When the co-cultured cells were treated with high glucose and metformin, a drug to treat diabetes, this combination improved the OCR and expression of glucose transporter proteins. Furthermore, combination of disease-relevant hPSC-derived tissues with other technologies, such as single-cell sequencing,^78^ would provide further insights.

## SC-islets as a functional cure

### Challenges of transplantation

Advances in primary human islet transplantation paved the way for transplant of their stem cell-derived counterparts. In humans, cadaveric islet transplantation previously required that patients use a combination of immunosuppressive drugs to prevent transplant rejection, including diabetogenic glucocorticoids, dampening the procedure’s therapeutic potential. Before 2000, only approximately 8% of allograft patients achieved insulin independence for more than 1 year.^79^ In 2000, Shapiro et al. published the Edmonton Protocol, which revolutionized islet transplantation by vastly improving the longevity of these allogeneic transplants. The authors attributed this to improved methods of islet isolation and the development of new immunosuppressive drugs, which allowed them to eliminate glucocorticoids in their islet transplantation protocol. All 7 patients in this landmark paper achieved insulin independence. A larger study used the Edmonton protocol and showed that 16 out of 36 subjects could demonstrate insulin independence for a year after final transplantation.^80^ Another study that tracked a cohort of transplant patients over 20 years showed that 32% of patients maintained insulin independence for 5 years, and 20% for 10 years after first transplantation.^81^

Though the development of the Edmonton protocol was a large stride forward in a viable treatment for T1D, failure to maintain insulin independence remains a challenge. One paper using positron-emission tomography and computed tomography (PET-CT) imaging and 18F-fluorodeoxyglucose (18F-FDG) labeling found that the expected radioactivity of 18F-FDG-labeled islets in the liver peaked at 75%.^82^ They also observed some radioactivity located in other parts of the body, and a sharp increase in C-peptide, which occurred within the first hour post-transplantation. The authors state that both of these observations corroborate that there is destruction of transplanted islets soon after transplantation.

Given the current state of SC-islet transplantation, studies to optimize the procedure and prevent SC-islet loss would be valuable to the field. One study investigating aspects of SC-islet transplantation, focusing on the site of injection, number of transplanted SC-islets, and diabetic state of mice, helped elucidate the impact of these factors on transplantation success.^83^ Regarding the location of the transplant, the authors observed that injection into the kidney capsule was the best location to lower blood glucose levels in diabetic mice. The authors also found that at least 2 million SC-islet cells were needed to reverse streptozotocin (STZ) induced diabetes and significantly increase C-peptide levels in immunocompromised mice. Interestingly, transplanting 5 million SC-islet cells underneath the kidney capsules of diabetic and nondiabetic mice led to similar serum human C-peptide levels when measured randomly at 2 weeks and via in vivo GSIS.

While the above study added to the body of literature optimizing the procedure of SC-islet transplantation, understanding challenges at the cellular level that may have led to graft failure is also important. Two main factors that are thought to contribute to graft death are oxygen deprivation and transplant rejection by the immune system.^84–86^ There may be value in the use of biomaterials or vascularization approaches to overcome the challenges of islet survival after transplantation.^87–92^ This review will focus on recent work improving SC-islet transplantation by reducing graft immunogenicity.

### Design of hypoimmune SC-islets

**Figure 3.**
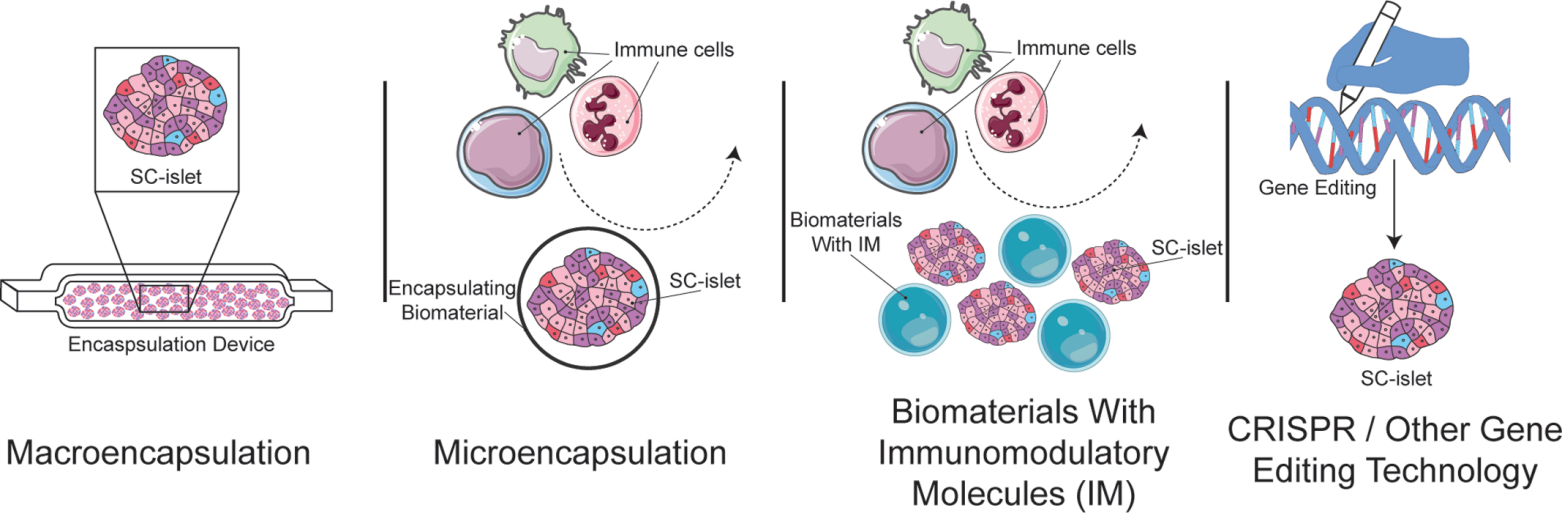
Strategies to achieve SC-islet immune protection. There are 4 main methods that have been investigated to protect SC-islets from the recipient immune system. One method uses a macroencapsulation device to contain the entire SC-islet transplant. Building on the idea of encapsulation, another method uses biomaterials to surround individual SC-islets—a technique called microencapsulation. This can include immunomodulatory molecules embedded into the encapsulating material to induce immune tolerance. A closely related method jointly transplants SC-islets with separate nonencapsulating biomaterials presenting the immunomodulatory molecules. The fourth method involves gene editing of SC-islets so that they themselves are immune evasive or not immunogenic.

The use of immunosuppressive drugs in islet transplantation has been previously mentioned in this review as a serious risk of islet transplantation.^84,93^ Patients taking immunosuppressants are at increased risk of malignancy and infections. Specific immunosuppressants such as calcineurin inhibitor tacrolimus, which was used in the 2000 Edmonton protocol paper, have been shown to be nephrotoxic. Current solutions for immunosuppression include the use of encapsulation devices or encapsulating biomaterials, combination approaches using encapsulation alongside immunomodulatory molecules, and genetic engineering to prevent immune rejection (Figure 3).

The encapsulation devices for islet transplantation are designed to contain islets and have pores that allow nutrients and waste products to diffuse through, but prevent immune cells from infiltrating the graft.^84,94,95^ The device also keeps the transplanted islets from leaving the graft. Keeping the graft within the device would allow for swift removal should the cells become malignant or the graft fibrotic, which has been a major problem when it comes to encapsulation devices. In 2015, a macroencapsulation device was reported to be able to maintain a graft of pancreatic endoderm cells that could mature into islet-like cells upon transplantation into mice.^96^ The encapsulated islet-like cells showed improved in vivo GSIS performance over time. Data from a phase I/II clinical trial building upon this technology reported that a number of patients receiving these encapsulated grafts had increased fasting C-peptide and post-meal C-peptide levels.^97^ Since then, many groups have reported success with islet macroencapsulation.^98–103^

An alternative to macroencapsulation is microencapsulation, in which biomaterials are used to encapsulate individual islets, improving nutrient exchange relative to macroencapsulation. In 2016, a group was able to show that SC-islets encapsulated in triazole–thiomorpholine dioxide alginate were able to maintain normoglycemia in diabetic immunocompetent C57BL/6J mice upon transplantation, and showed less immune cell infiltration 2 weeks post-transplantation.^104^ The incorporation of immunomodulatory molecules into biomaterials has paved the way for an alternate strategy that focuses on building immune tolerance to the islet transplant. One group investigated the ability of CXCL12 to enhance SC-islet microencapsulation.^105^ To do this, CXCL12 was mixed with alginate prior to SC-islet encapsulation. The authors found that encapsulation with CXCL12 improved SC-islet C-peptide secretion by in vitro GSIS. The encapsulated SC-islets containing CXCL12 were able to stably reduce the blood glucose of diabetic mice for a longer period of time than those encapsulated with alginate microcapsules alone. At 20 weeks post-transplantation, mice who had received encapsulated islets with CXCL12 had a significantly higher fasting serum C-peptide compared to that of mice that received encapsulated islets alone. Another group found that cotransplantation of islet allografts and polyethylene glycol hydrogel microspheres presenting a chimeric protein consisting of Fas ligand and streptavidin into diabetic nonhuman primates was able to decrease blood glucose levels.^106^ A preliminary study has investigated the use of human elastin-like recombinamers as an alternative to the aforementioned biomaterials in microencapsulation of human SC-islets.^107^

Taking the concept of immunomodulation one step further, genetic engineering of the islet cells themselves to prevent immune rejection is another strategy that has been investigated. For example, the transplantation of islet-like organoids overexpressing PD-L1 was able to improve blood glucose levels of NSG and diabetic immunocompetent C57BL/6J mice, similar to the transplantation of mouse islets.^108^ Another approach to genetically engineering protection from the recipient immune system is the deletion of human leukocyte antigens (HLAs). One group showed that deletion of all classical HLAs with the exception of HLA-A2 and HLA-E/F/G can reduce NK cell activation in response to SC-islet exposure in vitro.^109^ For 8 weeks, these deletions helped maintain the graft better than wild-type SC-islets when transplanted into NSG mice. Another group has shown the promise of combining HLA class I and II deletions with CD47 overexpression.^110^ They show that *B2m*^*−/−*^*Ciita*^−/−^ Cd47-overexpressing mouse and *B2M*^*−/−*^*CIITA*^−/−^ CD47-overexpressing human iPSC derivatives can be transplanted into allogeneic mice and humanized mice, respectively. The authors also observed that the gene-edited mouse and human iPSCs and iPSC derivatives grew or maintained their volume when compared with injected wild-type counterparts. These cells also reduced NK cell activation in vitro, as evidenced by decreased signal on IFNγ Elispot assays. These findings have been more robustly supported with data from transplantation of human SC-islets and rhesus macaque primary islets with the aforementioned gene edits into diabetic immunocompetent humanized mice^111^ and nonhuman primates respectively.^112,113^

While the rationale for the above genetic edits came from prior literature, there have been efforts to do genome-wide screens using CRISPR/Cas9 to conduct an unbiased search for genes that would be protective against the immune system. Two papers have reported results from in vivo CRISPR knockout (CRISPRko) screens. A genome-wide CRISPRko screen in which CRISPR library-transduced SC-islets were transplanted into a humanized mouse model identified CXCL10 as a knockout target to support graft survival and function.^114^ This cytokine is thought to play a role in recruiting T cells and macrophages in T1D pathogenesis. Another group conducted a CRISPRko screen by transplanting CRISPR library-transduced NIT-1 mouse insulinoma cells into nonobese diabetic (NOD) mice. They found that RNLS, a protein that has been shown to help regulate blood pressure and heart rate, can reduce CD8 T-cell activation and improve graft survival.^115^

## Concluding statements

Protocols to produce SC-islets are designed to recapitulate the endogenous developmental process in humans. Multiple different protocols exist each creating functional SC-islets that mimic their primary counterparts. Single-cell and snATAC sequencing have revealed cell type-specific transcriptional and chromatin differences between primary and SC-islets. In particular, a cell type not found in primary human islets, SC-EC cells, was discovered through sequencing methods. Further studying of this off-target cell type could lead to an islet composition more reminiscent of primary islets. In addition, studies have found SC-islets to be immature. In vivo transplantation and circadian rhythm modification have been shown to improve maturation.^26,83,116^ Advancements in understanding and improving SC-islets will lead to progress in disease modeling and as a cell therapy for diabetes.

This review summarizes recent publications using SC-islets to both study diabetes and develop strategies to protect transplanted islets. To study diabetes, groups have differentiated patient-derived iPSCs into SC-islets or used CRISPR/Cas9 to study specific mutations that cause different forms of diabetes. In addition, compounds have been added to SC-islet media to mimic diabetic environments. SC-islets are not the perfect model system; however, as the protocols improve, so will disease modeling. Though SC-islets are still in need of optimization, the cumulative efforts thus far have demonstrated the vast potential of these cells for transplantation.^93^ However, protecting SC-islets from the recipient immune system remains a challenge. Vertex Pharmaceuticals has 2 ongoing phase I/II clinical trials (NCT05791201 and NCT04786262) using SC-islet transplantation to treat T1D in a small number of patients.^117^ These efforts are timely, as the United States Food and Drug Administration has recently approved the use of primary pancreatic islets as a cell therapy for T1D. In summary, SC-islets have a multitude of applications, and improvements to islet identity, function, and transplantation will advance the study of diabetes and its treatment.

## Data Availability

No new data were generated or analyzed in support of this research.
